# An evaluation of a juvenile idiopathic arthritis retreat for families

**DOI:** 10.1186/s12969-015-0010-3

**Published:** 2015-04-21

**Authors:** Michelle L Burbage, Meredith B Mason, Laura A Nabors, Jessica C Kichler

**Affiliations:** Health Promotion & Education, School of Human Services, College of Education, Criminal Justice, and Human Services, University of Cincinnati, PO Box 210068, ML 0068, 526TC, Cincinnati, OH 45221-0068 USA; Behavioral Medicine and Clinical Psychology, Cincinnati Children’s Hospital Medical Center, Cincinnati, USA

**Keywords:** Juvenile Idiopathic Arthritis, Arthritis, Family camp, Family retreat, Children, Coping strategies, Support, Education

## Abstract

**Background:**

The current study evaluated the support and education families with a child who has Juvenile Idiopathic Arthritis (JIA) received during a weekend family retreat.

**Methods:**

Thirty-one parents completed surveys at the end of the retreat session. Survey questions examined parent’s perceptions of the family retreat experience including what they learned and how beneficial it was to their family.

**Results:**

Results indicated that the family retreat was beneficial to both the children with JIA and their siblings. Children with JIA had the opportunity to see that other children have this disease as well. At the family retreat, siblings were provided the chance to see that children with JIA are capable of many accomplishments. In addition, the family retreat provided opportunities to learn from other families and offered families reduced isolation.

**Conclusions:**

The family retreat was successful in delivering education and support to families, which might not otherwise be obtained via a clinical setting. Parents learned how to support their child with JIA, develop their child’s pain management skills, and remain positive as a family. Future research should assess if the education and support family retreats provide have long-term improvement to managing and coping with JIA.

## Background

### Juvenile idiopathic arthritis retreat for families

Juvenile Idiopathic Arthritis (JIA) affects 294,000 children in the United States ‘[1]. These children not only suffer from this disease, but comorbid emotional concerns as well. Family retreats providing education and support may help to relieve both the JIA patient and their families of emotional distress. Children with JIA may have lower levels of health related quality of life (HQOL) and self-esteem. They may also experience social isolation, depression, and anxiety [2]. Bomba et al. [3] found that children suffering with JIA often experienced reduced psychological maturity and reported low levels of well-being. Additionally, parents of children with JIA may experience depression and anxiety related to their child’s chronic illness [4]. Parent and child emotional reactions may be interrelated when a child has JIA [5]. Hence, both children and parents of children with JIA experience needs for support and education about the disease. These needs may not be met solely by education from the medical team [5].

Various opportunities for patients and their parents to meet with other families, who are also coping with JIA, are methods for assisting parents and children in coping with the disease [6]. Family retreats play a major role in enhancing family functioning and in offering opportunities to gather further information about the child’s disease [7,8]. Specifically, family retreats for chronically ill children provide families additional education, coping strategies, and resources, which cannot be met in the clinic or be taught by the medical team [8]. Barlow and Ellard [9] found that psychosocial interventions for JIA, including family retreats, provide families this type of knowledge. These researchers found that after attending family retreats, children reported reduced pain as well as improved self-esteem and adherence to their treatment regimens [9]. Additional research evaluating the impact of family retreats for children with JIA and their families is still needed. The current study presents results from an evaluation of a weekend family retreat designed to educate and provide support for families with a child who has JIA as well as assess parent perceptions. More specifically, the following research questions were examined: 1) Do parents perceive participation in the JIA family retreat to be helpful? 2) Do parents find the retreat to be enjoyable? 3) Do parents perceive the retreat to be beneficial to the siblings of children with JIA? 4) Do parents perceive the retreat to be beneficial to children with JIA? 5) What did the parents learn from attending the retreat?

## Methods

### Participants

Thirty-one parents completed surveys at the close of the family retreat session (100% response rate). Thirteen were fathers and 18 were mothers. All were Caucasian. Twenty-two children with JIA, ranging from 7 to 18 years of age attended the family retreat with their parents. Fifteen siblings also attended the family retreat with their families.

### Family retreat activities

The family retreat intervention consisted of lectures and discussions to boost parent, child and sibling knowledge of JIA and how to cope with a child’s JIA as a family team. Lectures were used to review treatments to assist children in coping with pain flares and fatigue. The children and his or her family members met with others in small groups to develop family plans to enhance child and family coping. Staff from the local Arthritis Foundation ran groups and provided brochures and materials. They stressed family-level planning to improve the child’s involvement in school and extra curricular activities. In addition, families participated in camp-like activities, such as a ropes course, hiking, and cycling, which were part of routine camp activities. These activities were designed to build each family member’s self-esteem and to foster a sense of group cohesiveness.

### Procedures

A university-based institutional review board approved this study. Consent was required for study participation. Parents completed surveys independently and were informed that discussion of negative and positive impressions were welcomed as their responses were valuable to quality improvement efforts. One of the authors supervised data collection. A team of three research assistants played games and interacted with children so that parents had time, without interruptions, to record their answers. No identifying information was included in the surveys, ensuring patient anonymity.

Parents responded to closed and open-ended survey questions examining their impressions of their family retreat experience. Questions included open-ended items indicating parents’ perceptions of how helpful the family retreat was, what they hoped to accomplish by bringing their family to the retreat, and what they learned during the family retreat. Parents responded to “yes,” “no,” or “don’t know” questions about whether they believed the family retreat was beneficial for siblings and whether having a child with JIA in the family changed family life. After providing a response to these questions, parents then provided reasons for their responses. They also responded to two questions about how much they liked the family retreat and how much the family retreat helped their child with JIA on a 6-point Likert scale (6 = “great” to 1 = “very poor”).

### Data analyses plan

Qualitative methods were used to analyze qualitative data provided by parents. A team of three researchers examined parent responses to qualitative questions. An open coding approach using memos was used to determine relevant categories that emerged in the data and could be classified as themes representing parent responses [10,11]. After this initial assessment, a second review of the data was performed. The coders identified themes in parents’ responses and representative quotes for each theme were selected. Over a period of three meetings, each researcher reviewed all data and a criterion of 100% agreement was used to determine themes in the data. After themes were selected, the team of researchers identified representative quotes for each theme. Information was then conveyed to two staff members at the local Arthritis Foundation who conducted the camp. This triangulation process confirmed findings.

## Results

Twenty-three of the parents (74%) reported that having a child with JIA in the family changed how things were done in the family. The most common response for these family changes was due to the child’s mobility limitations. One mother indicated the magnitude of the effect this illness has on her family and how healthcare needs encompasses their lives. The mother wrote, “where we live-health insurance we have-effect most aspects of our life.”

Seventeen of the parents (55%) said the family retreat was “great” (the highest score on the six-point scale) and thirteen (42%) of the parents reported that camp was “good” (a five on the six point scale) in terms of helping the child with JIA because he or she was exposed to other children and families. Twenty-four (77%) of the 31 parents reported that siblings without JIA who attended the family retreat also benefitted from participating. Three themes were discovered among the responses provided by parents for the siblings of children with JIA. Specifically, they often felt that their children who did not have JIA benefited by seeing other families with a child who had JIA and were able to see that “kids with arthritis can participate in a lot of things.” Another theme was enjoyment as parents felt siblings enjoyed the retreat. The parents felt that their children who did not have JIA also had an opportunity to learn how JIA affects other young people, as most of the children with JIA only had experience with their own sibling.

Parents responded to a general question indicating whether they felt that participating in the family retreat was helpful. In general, parents reported their families benefited from the supportive and educational family retreat and the camp setting. Specifically, 30 parents reported the family retreat was helpful, and only one parent thought that participating in the family retreat was not helpful to their family. Parents also provided written information about ways in which the family retreat was helpful. The themes and representative quotes from parent surveys about the ways in which participating in the family retreat was helpful are presented in Table 1.

**Table 1:** **Reasons that family retreat participation was helpful**

**Themes**	**Examples of representative quotes from parents**
Strengthened family as a unit	Mother – “Yes it brought us together and we were able to focus in a positive way on our daughter and each other.”
Helps child with JIA understand he or she is not alone – there are others out there like me	Mother – “Yes! It made us see that there are other kids out there like my daughter.”
	Mother – “Yes! My daughter’s illness has affected our entire family. By spending the weekend with other families, we have seen that we are not alone, while living with this disease.”
	Father – “Yes. These types of opportunities allow my daughter to see that there are other kids like her. It allows my sons to also see that other kids deal with this disease and that other siblings deal with it as well.”
Other family members learned more about the child’s disease	Father – “Yes, it helps other siblings understand what their sister is going through.”
Opportunities to learn from other families	Mother – “Helpful for us to meet other families and brainstorm various solutions to problems.”
	Father – “Absolutely! We all need to hear the messages and learn how other families are dealing with arthritis.”

Qualitative coding of the survey information provided by parents indicated that four themes emerged: (1) the family retreat experience strengthened the family as a unit; (2) the experience helped the child with JIA understand that he or she is not alone and there are other children struggling with this disease; (3) the experience promoted understanding of the child with JIA’s experience for other family members; and (4) the experience provided opportunities/chances to learn from other families coping with similar issues.

The most important theme that emerged appeared to be parent reported increase in support and reduction of isolation as a result of being at the family retreat. For example, one mother reported that her family was feeling less isolated after participating in the family retreat because, “It made us see that there are other kids out there like my daughter.” Another mother echoed this sentiment stating that, “My daughter’s illness has affected our entire family. By spending the weekend with other families, we have seen that we are not alone, while living with this disease.”

The only drawback to family retreat participation was that some of the camp activities (e.g., ropes exercises) were physically challenging for children with JIA and parents worried that their child could feel a lack of accomplishment if he or she could not participate in some of the activities along with all of his or her family members.

Ninety-seven percent of parents were satisfied with their family retreat experience. Parents’ responses about what they learned at the family retreat centered on four themes, which are presented in Table 2.

**Table 2:** **Parental responses about what they learned at camp**

**Themes**	**Examples of representative quotes from parents**
Learned how to encourage, support, and recognize my child	Father – “We learned how to work together to encourage and help our son (brother) to accomplish difficult tasks”
	Mother – “To remember to encourage him to always do his best”
	Mother – “It is okay to push her + stop so much over protecting”
Pain management	Mother – “The discussion [discussion] on pain management was excellent!”
	Mother – “Some pain management skills + things to talk about”
	Father – “The presentation on pain management from a psychological perspective was helpful”
Social support	Father – “It helps to understand how other parents cope”
	Mother – “We know the Arthritis Foundation is always there to support us”
	Mother – “Just being with other families who are in the same shoes”
Staying positive/Encouragement	“We learned to try our best and encourage each other with Alpine Tower”
	“Encouragement and positive support and feedback”
	“How to encourage and cheer for not only your family but others. Everyone is in this together. Not to the same extent, but still the same”
	“To stay positive”
	“How to get along together”
	“I think my family learned to be more patient with each other”

It was evident that the supportive, group aspect of the family retreat experience was beneficial for the parents in improving their knowledge about how to help and support their child with JIA. Parents also enjoyed the lecture on pain management for their children with JIA and felt it was very beneficial. The family retreat helped with tips for helping the family to stay positive while a child was coping with disease-related limitations.

## Discussion

Results of this study indicated that parents who attended the JIA family retreat perceived that the event was helpful in providing social and educational support to all members of their families. These findings were consistent with research showing that family retreats are successful in providing information, resources and support to families [8]. The results also indicated that parents thought that the retreat was beneficial to siblings of children who have JIA as it offered them the opportunity to learn how to cope from observing and learning directly from other families. Past research has similarly found that families with a child with JIA can support one another [6].

Findings from past studies show that family retreats can enhance family functioning and aid families in developing coping strategies [7,8]. The results from the present study correspond to these findings. In the present study, parents also learned how to support their child with JIA, develop their child’s pain management skills, and stay positive as a family via the family retreat. The support found at the retreat for the parents, the siblings, and the children with JIA was an invaluable feature of a group experience that is difficult to obtain in one-on-one settings, such as follow-up appointments in a medical clinic.

The main strength of the study was that data was collected in order to assess the usefulness of a family retreat as an intervention to improve quality of life for patients and families with JIA. While the concept of a JIA retreat is not new, this particular retreat improves upon previous JIA family retreats since the model for evaluating camp was based on a thorough review of evidence-based literature and expert review. Survey questions were developed based on a literature search as well as from a series of interviews with staff that have participated in camps at a local Arthritis Foundation.

There are factors that may have limited the generalizability of the present study findings. Data was self-reported by parents in the family, thus some individuals may have provided socially desirable responses. Also, all retreat participants were Caucasian thus limiting the generalizability of the findings for other races and ethnicities. In addition, the children with JIA and their siblings were not directly assessed for their perspectives on the family retreat. Moreover, exact diagnostic information in regards to the types of JIA patients who attended the retreat was not collected. Thus, it is unclear if certain types of JIA among the children impacted parent perceptions. Future longitudinal studies assessing the families’ social and educational improvement due to participating in a family retreat is needed. Moreover, future research can assess whether retreat costs impacted parent perceptions of the retreat. Future studies can further assess if the education and support family retreats provided families with a child who has JIA has long-term improvements to health outcomes and coping with the disease.

## Conclusions

The present study found the JIA family retreat to be beneficial to parents, siblings, and children with JIA. It is important that children with JIA and their families receive support and are assisted in improving family functioning as well as coping strategies. Thus, it is essential that other outlets, such as family retreats, be offered to families with this health condition. As opposed to clinical settings, the group setting of the retreat can aid in fostering support from other families who also have a child with JIA. Additional research assessing health outcomes due to attending family retreats is warranted.
